# A scale CUBIC-based clearing protocol preserves fluorescence in mouse rib bone and cartilage

**DOI:** 10.1093/jbmrpl/ziag122

**Published:** 2026-07-28

**Authors:** Mackenzie Fernandez, Emerson Soo-Hoo, Kaelynn Perez Castro, Kimtee Kundu, Divya D Patel, Francesca V Mariani

**Affiliations:** Department of Stem Cell Biology and Regenerative Medicine, Keck School of Medicine of USC, Los Angeles, CA 90033, United States; Department of Stem Cell Biology and Regenerative Medicine, Keck School of Medicine of USC, Los Angeles, CA 90033, United States; STAR Program, Keck School of Medicine of USC, Los Angeles, CA 90033, United States; Department of Stem Cell Biology and Regenerative Medicine, Keck School of Medicine of USC, Los Angeles, CA 90033, United States; STAR Program, Keck School of Medicine of USC, Los Angeles, CA 90033, United States; Department of Stem Cell Biology and Regenerative Medicine, Keck School of Medicine of USC, Los Angeles, CA 90033, United States; STAR Program, Keck School of Medicine of USC, Los Angeles, CA 90033, United States; Department of Stem Cell Biology and Regenerative Medicine, Keck School of Medicine of USC, Los Angeles, CA 90033, United States; STAR Program, Keck School of Medicine of USC, Los Angeles, CA 90033, United States; Department of Stem Cell Biology and Regenerative Medicine, Keck School of Medicine of USC, Los Angeles, CA 90033, United States

**Keywords:** optical clearing, bone, cartilage, transgenic mouse, growth plate, bone repair

## Abstract

Tissue clearing has emerged as a critical method for observing fluorescent proteins in animal models. This technique allows for imaging by making tissues transparent while preserving the natural structure of biological samples and removing the need for labor-intensive tissue sectioning. Conventional tissue clearing methods can be classified into three primary types: hydrophobic, hydrophilic, and hydrogel-based, each possessing unique benefits and drawbacks. Hydrophobic techniques, like benzyl alcohol-benzyl benzoate (BABB), 3D imaging of solvent-cleared organs (DISCO), and polyethylene glycol associated solvent system (PEGASOS), create highly clear samples but frequently lead to tissue contraction and may lead to diminished fluorescence. Hydrophilic techniques, such as the Clear, Unobstructed Brain/Body Imaging Cocktails (CUBIC) series, provide improved preservation of tissue integrity but might fall short of achieving complete transparency. Hydrogel-based methods such as Clear Lipid-exchanged Acrylamide-hybridized Rigid Imaging/Immunostaining/In situ hybridization-compatible Tissue-hYdrogel (CLARITY) and Entangled Link-Augmented Stretchable Tissue-hydrogel (ELAST) preserve biomolecules, but the protocols may be intricate and expensive. Clearing skeletal tissues such as bone and cartilage can be especially difficult because of their dense extracellular matrix, which may have abundant collagens, high mineral composition, and dense obscuring bone marrow (BM). In addition, clearing procedures frequently do not provide adequate transparency without damaging proteins that fluoresce. To overcome these constraints, we created a clearing protocol based on published Scale CUBIC techniques to be used on adult mouse rib bones. Our method does not entail decalcification and improves transparency in about 48 h. Utilizing transgenic mice, we show that this method effectively maintains both endogenous fluorescence and tissue morphology. This enhanced approach offers a dependable technique for imaging hard tissues such as rib bones in transgenic mouse models.

## Graphical Abstract

**Graphical Abstract:**
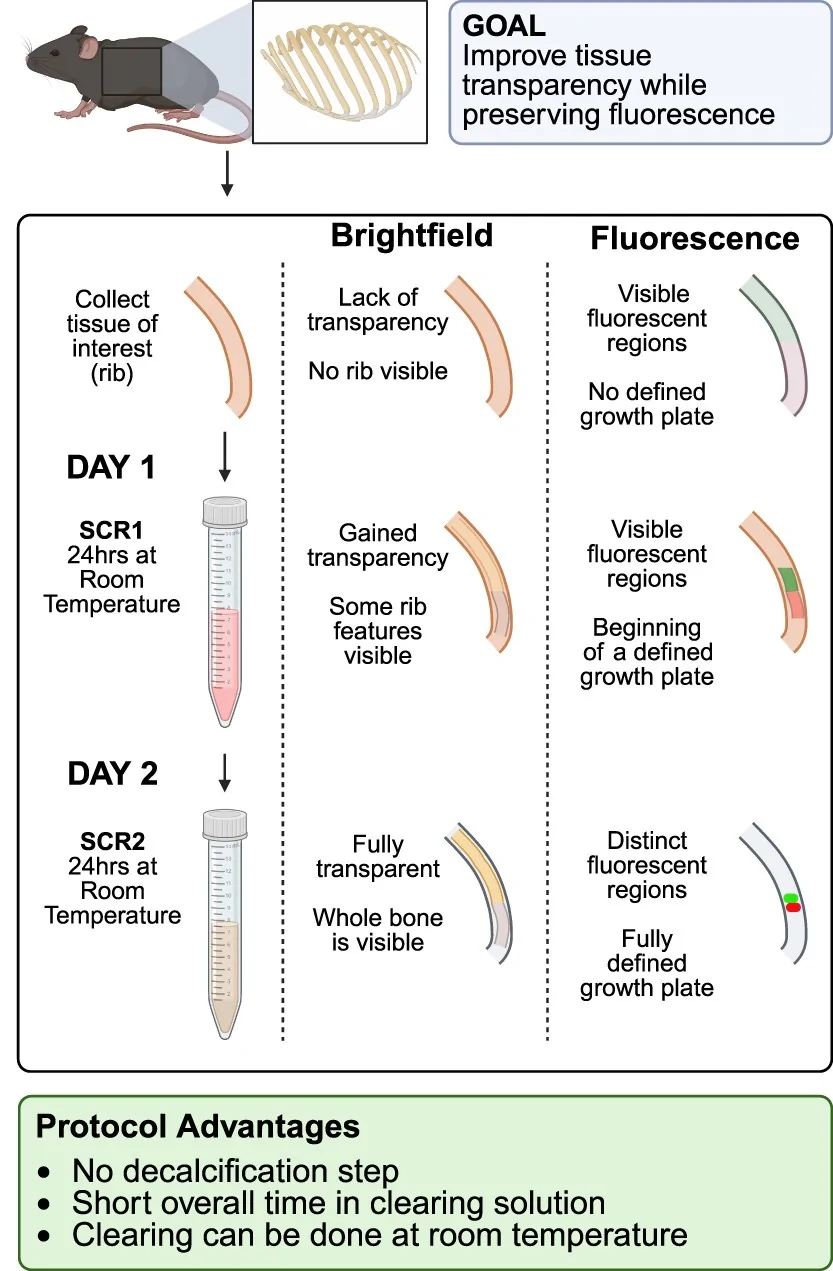

## Introduction

The increasing use of transgenic animals expressing fluorescent reporter proteins to understand skeletal biology has heightened the need for effective imaging and analysis methods. While tissue sectioning is widely used because it allows for high-resolution imaging and protein fluorescence can be preserved through cryosectioning,^1^ it inherently limits the ability to visualize features within entire tissues. Optical clearing techniques offer a complementary approach by increasing tissue transparency through various chemical or physical treatments, thus facilitating deeper and more comprehensive imaging of whole tissue samples. Our goal here was to develop an optical clearing method that maintains the natural tissue architecture, reduces processing time compared to existing methods, and minimizes the loss of fluorescence from endogenous proteins. Optical clearing protocols operate by manipulating the refractive index (RI) of tissues. Light scattering within biological samples arises from RI mismatches; therefore, tissue clearing aims to minimize these mismatches by selectively removing intracellular and extracellular components through specialized clearing reagents.^2^

Numerous tissue clearing protocols have been developed, each with their own specific reagents and timelines. Tissue clearing methods are generally classified into three major categories: hydrophobic (organic solvent-based), hydrophilic (aqueous reagent-based), and hydrogel-based approaches. Hydrophobic clearing methods typically require tissue dehydration and result in a high final RI (RI > 1.54). Common examples include benzyl alcohol-benzyl benzoate (BABB),^3^ Three-dimensional imaging of solvent-cleared organs (DISCO),^4^ and polyethylene glycol associated solvent system (PEGASOS).^5^

In contrast, hydrophilic clearing methods yield lower final refractive indices RI (~1.4 to 1.52) and use aqueous-based reagents. Because hydrophobic methods use dehydration, samples often undergo expansion or contraction, which can alter tissue dimensions and optical properties. Hydrophilic methods, such as those in the Clear Unobstructed Brain/Body Imaging Cocktails (CUBIC) series, largely avoid these issues and preserve tissue size and morphology.^6^ One iteration of the CUBIC series is the Scale CUBIC protocol. The term Scale reflects the protocol’s ability to render large biological samples optically transparent while preserving their original size and structural integrity, enabling imaging across spatial scales without physical sectioning. Scale CUBIC was initially developed and tested in brain tissue and requires only two reagents: Scale CUBIC-1 and Scale CUBIC-2. Scale CUBIC-1 (SCR1) promotes tissue transparency primarily through lipid removal and protein hydration, driven by high concentrations of urea and detergents that reduce light scattering. Scale CUBIC-2 (SCR2) subsequently performs RI matching, further minimizing optical heterogeneity within the tissue. Using this two-step approach, the protocol achieved high transparency of a whole mouse brain in approximately 14 d.^7^

The third category, hydrogel-based tissue clearing, involves embedding of tissue samples in a hydrogel matrix that physically or chemically stabilizes biomolecules in place. Benefits of this strategy include increased durability of samples and hydrogel expansion that leads to better spatial resolution with light microscopy. Representative hydrogel-based methods include Clear Lipid-exchanged Acrylamide-hybridized Rigid Imaging/Immunostaining/In situ hybridization-compatible Tissue-hYdrogel (CLARITY) and Entangled Link-Augmented Stretchable Tissue-hydrogel (ELAST).^2^^,^^8^

### Development of the protocol

Complex hard tissues, such as bones and teeth, present unique challenges for optical clearing compared with soft tissues. These tissues are often highly mineralized, rich in collagen, and contain pigmented BM, features that render many clearing techniques developed for soft tissues ineffective or incompatible.^9^ When adapting clearing protocols for hard tissues, the inherent limitations of the three main categories of clearing methods may need to be considered. Hydrophobic (solvent-based) methods require dehydration, which leads to tissue shrinkage and loss of fluorescence. Hydrophilic (aqueous-based) clearing protocols better preserve fluorescence, but frequently fail to achieve sufficient transparency of dense, mineralized tissues. Finally, while hydrogel-based clearing strategies protect biomolecules from harsh chemical treatments and maintain tissue architecture, they are comparatively complicated, time-consuming and expensive. To address these challenges, specialized approaches for hard-tissue clearing include methods within the DISCO series, PEGASOS, and Bone CLARITY.^4^^,^^5^^,^^10^ In general, successful clearing of hard tissues has included the following steps—perfusion/fixation, decalcification, decolorization, delipidation, and RI matching.

Our protocol builds upon the previously established Scale CUBIC reagent framework^5^^,^^6^ and has been specifically curated to retain native fluorescence from GFP and mCherry reporter proteins. Green fluorescent proteins such as GFP are widely recognized for their robustness and photostability across diverse imaging conditions, making them relatively resistant to quenching and photobleaching compared with many red fluorescent proteins including mCherry. In contrast, differences in spectral properties and environmental sensitivity render mCherry more susceptible to signal loss under conditions where GFP fluorescence remains detectable.^11–13^ Despite this limitation, GFP and mCherry remain attractive fluorescent proteins for combinatorial use due to their well-separated emission spectra, which allow for efficient multichannel imaging and minimal spectral overlap.^14^ Accordingly, optimizing tissue clearing conditions to retain fluorescence from both reporters could be of particular value.

We compared several established clearing approaches, including PEGASOS, Stabilization under Harsh conditions via Intramolecular Epoxide Linkages to prevent Degradation (SHIELD), and Clearing-Enhanced 3D Microscopy (Ce3D) to our Scale CUBIC method.^15^^,^^16^ A key difference between our protocol and other methods is the elimination of the decalcification step which allows for better retention of our fluorescent markers. Additionally, our protocol improves sample transparency and appropriate RI matching in a substantially shorter timeframe, requiring approximately 48 h for completion. Notably, we found that relatively minor modifications to a published Scale CUBIC protocol can have a pronounced impact on fluorescence preservation.^14^

To evaluate performance, we applied the protocols to rib bone and cartilage tissue from a dual-reporter mouse model carrying *Col10-mCherry* that allows for visualization of hypertrophic chondrocytes, and *Col1(2.3)-GFP* that allows for detection of developing osteoblasts (Tg[Col10a1-mCherry]3Pmay, Jackson Labs Strain #:017465, B6.Cg-Tg[Col1a1*2.3-GFP]1Rowe/J, Jackson Labs Strain Strain #:013134).^17^^,^^18^ Animals were perfusion-fixed with paraformaldehyde (PFA) and tissues were subsequently stored in 1× PBS at 4 °C prior to clearing.

NOTE: All procedures are in accordance with an animal protocol approved by the Institutional Animal Care and Use Committee at the University of Southern California. Animals were housed in a barrier facility and provided food and water ad libitum.

## Materials and methods

### Equipment

- Magnetic stir bars
- Stirring hot plate
- Balance, accurate to at least 0.1 g
- Appropriate microscope (depending on goal of project) with fluorescent imaging capacities

### Reagents and supplies

- Paraformaldehyde (PFA), Thermo Scientific (cat. 28 908)
- 1× PBS
- Urea, Sigma (cat. U5378-100G)
- Quadrol, VWR/TCI America (cat. TCT0781)
- Triton X-100, Sigma (cat. ×100)
- Sucrose, VWR (cat. 97 061-428r)
- Triethanolamine, Fisher Scientific (cat. T4071)
- Glass-bottomed dishes for imaging, Cellvis (cat. D35-20-1-N)

## Reagent setup

Solutions are prepared as described by Susaki et al.,^7^ with two key modifications: the degassing step was omitted, and SCR2 is formulated with 2% instead of 10% triethanolamine to better preserve mCherry fluorescence. Additionally, sample clearing is performed at room temperature. Higher temperatures, although improving overall tissue transparency, caused substantial loss of fluorescence signal. In addition, we found that if we used Quadrol purchased from Sigma (cat. 122 262), the clearing solutions would tend to become cloudy over time and sometimes cause reagents to crash out of solution. Using Quadrol purchased from TCI (cat. TCT0781) improved this problem.

Scale CUBIC Reagent 1 (SCR1) components are urea (25%), Quadrol (25%), distilled water (35%), and Triton X-100 (15%) by weight percentage. Measure Quadrol, water, and Triton X-100 using a beaker and balance. Combine all into a clean autoclaved glass solution bottle. Heat the bottle on a hot plate to 60 °C, using a stir bar to mix slowly at 500 rpm (note that higher temperatures can result in urea degradation, which will reduce the clearing capacity of the solution). Once dissolved and mixed, remove the bottle from the hot plate and add the rest of the water which will cool the mixture. Once cooled to 32-37 °C, mix in the Triton X-100, using a magnetic stir bar. SCR1 can be stored at room temperature and used for up to 3 mo.

Scale CUBIC Reagent 2 (SCR2) is composed of urea (25%), sucrose (50%), distilled water (15%), and triethanolamine (2%) by weight percentage. Measure urea and sucrose using a weighboat and balance; and measure distilled water and triethanolamine using a beaker and balance. Combine urea, sucrose, and distilled water into an autoclaved bottle. Heat the mixture on a hot plate, while gently stirring with a magnetic stir bar until fully dissolved. Allow the solution to cool to room temperature, then add triethanolamine and stir until completely mixed. SCR2 can be stored at room temperature and used for up to 3 mo.

## Procedure

### Whole mouse perfusion and storage

While under isoflurane-induced anesthesia perfusion-fix the transgenic mouse with 1× PBS and then with 4% PFA following a standard protocol.^19^^,^^20^ The fixed tissue can be stored in 1× PBS at 4 °C until clearing.

### Sample collection

Surgically remove the tissue/region of interest (rib) from the mouse.

### Sample clearing

1. Place tissue sample in a polypropylene tube (15 mL, VWR cat. 89 039-666; polystyrene is compatible with this protocol as well). Immerse in 10× volume (by weight of the sample) of SCR1. Protect the tube from light (ie, wrap in foil). Leave the sample in SCR1 at room temperature (20-22 °C) for 24 h.
2. Remove the SCR1 from the tube and replace with SCR2 until volume is 10× that of the sample. Leave the sample in SCR2 at room temperature (20-22 °C) for 24 h.
3. Replace with fresh SCR2.

### Sample imaging

1. Remove sample from the tube with forceps and submerge in SCR2 for imaging.
2. Lower magnification images can be collected using bright field and epifluorescence imaging (compound or stereoscope). Higher magnification images are best collected using a Leica Thunder or Confocal imaging system. Samples (submerged in SCR2) can be imaged through a glass-bottomed dish with inverted objectives (eg, Cellvis cat. D35-20-1-N).

### Sample storage

After imaging, the sample can be returned to the tube and stored in SCR2 at room temperature (20-22 °C). Storing at 4 °C can result in solidification of the solution and is not recommended.

### Protocol notes

Susaki et al.^7^ previously described incubating samples in solutions at 37 °C, with some steps requiring gentle shaking or rotation. We have found that samples achieve transparency and retain strong fluorescence at room temperature and no shaking or rotation is required. Additionally, we have found that reducing triethanolamine from 10% to 2% was key to retaining mCherry fluorescence. Reducing the triethanolamine concentration is expected to slightly lower the RI of SCR2 relative to the reported value of 1.48, while maintaining sufficient index matching for effective optical clearing.^7^

The most critical step of this protocol is the accurate calculation and mixing of the SCR solutions. Incorrect triethanolamine concentration can result in crystallization of the entire SCR2 solution. It is also essential that the SCR2 solution is fully cooled before adding the triethanolamine. Inadequate mixing or excessive heat can cause cloudiness or incomplete dissolution of the reagents.

The optimized timeline for this protocol is 48 h, with samples immersed in each solution for 24 h. Multiple trials were conducted using different immersion timings for SCR1 or SCR2. Imaging of these samples revealed marginal differences between trials, demonstrating that 24 h of immersion per solution is sufficient for effective clearing rib samples.

### Quantitative analysis

#### Clearing efficiency

To assess clearing efficiency, we developed a grid-visibility assay. Samples were placed directly on a high-contrast printed grid and imaged before and after clearing using the Nikon AZ100 macroscope and a Leica K3C camera.

Images were converted from Red, Green, Blue (RGB) to CIELAB (Commission Internationale de l’Éclairage (International Commission on Illumination), L: perceptual lightness, A: green–red axis, B: blue–yellow axis) color space in the image analysis package Fiji (ImageJ), and the L* (lightness) channel was extracted for analysis. Conversion to CIELAB was performed to separate luminance information from color information and to provide a perceptually uniform measure of brightness. Because tissue transparency primarily affects light transmission rather than color, the L* channel was used as a quantitative measure of grid visibility. Rectangular ROIs were manually defined, and the width dimensions were matched to the width of the grid lines. Mean L* intensity values were measured for each ROI over the grid line and at an adjacent location, and a grid visibility index (GVI) was calculated as the ratio of the mean L* value of the background region to the mean L* value of the grid line region:


$$ \mathrm{GVI}=\frac{L\mathrm{background}}{L\mathrm{grid}\ \mathrm{line}} $$

Grid visibility indices were determined for each sample before clearing (GVI_before) and after clearing (GV_after). The same measurements were performed on unobstructed grid regions to obtain the reference index (GVI_grid). Clearing efficiency was normalized to the unobstructed grid reference according to:


$$ \mathrm{Clearing}\kern0.17em \mathrm{efficiency}\;\left(\%\right)=\frac{\left(\mathrm{GVI}\_\mathrm{after}-\mathrm{GV}\_\mathrm{before}\right)}{\left(\mathrm{GVI}\_\mathrm{grid}-\mathrm{GV}\_\mathrm{before}\right)}\times 100 $$

where GVI_grid represents the maximum observable grid contrast measured in the absence of tissue. Thus, 0% corresponds to the uncleared sample and 100% corresponds to the maximum observable grid contrast. Higher values indicate greater optical transparency and more effective tissue clearing. The measurements shown are intended as an illustrative complement to the visual observations over the course of the protocol rather than a statistical comparison across biological replicates.

#### Signal to background ratios

To assess fluorescence preservation during tissue clearing, GFP and mCherry protein fluorescence was quantified from representative images acquired at selected stages of the clearing protocol. Fiji (ImageJ) was used to split out the color channels and ROIs were manually defined within areas exhibiting specific GFP or mCherry fluorescence or in areas expected to be exhibiting signal based on the anatomy (cartilage and bone). Mean fluorescence intensity was then measured for each ROI in each channel. Background fluorescence was measured from adjacent regions within the same image where signal was not expected (connective tissues and muscle).

Fluorescence preservation was expressed as a signal-to-background ratio (SBR), calculated as:


\begin{align*} &\mathsf{Signal}-\mathsf{to}-\mathsf{background}\ \mathsf{ratio} \\ &=\mathsf{Mean}\ \mathsf{signal}\ \mathsf{intensity}/\mathsf{Mean}\ \mathsf{background}\ \mathsf{intensity} \end{align*}

where mean signal intensity corresponds to the average fluorescence intensity within reporter-positive regions and mean background intensity corresponds to the average fluorescence intensity within adjacently defined non-fluorescent regions. For time-course analyses, measurements were obtained from representative samples at d 0, 1, 2, and 14 of the clearing protocol. For comparisons of decalcification conditions, similar measurements were performed on representative images from the Scale CUBIC, EDTA plus Scale CUBIC, and RapidCal plus Scale CUBIC treatment groups. These measurements were used to assess the retention of tissue-specific fluorescent signals and the extent of diffuse background fluorescence following tissue clearing and decalcification.

### Confocal image processing and 3D rendering

Z-stacks acquired from a Leica SP8X Confocal were imported into Fiji (ImageJ) for visualization and processing. A composite stacked image with 2-channels was then generated. A maximum intensity projection (MIP) was created using the Fiji Z-Projection function, in which the maximum fluorescence intensity value along the *z*-axis was projected into a 2D image; rendering spanned approximately 300 μm in depth. To generate a 3D rendering from the composite stack, image noise was reduced using the Despeckle and Smooth filters and edge definition was enhanced using an unsharp mask filter. Brightness and contrast adjustments were applied uniformly across the image stack to improve visualization. Three-dimensional volume renderings were then generated from the processed image stacks using the Fiji 3D Viewer plugin with a sampling factor of 1. Brightness and contrast were adjusted during rendering to optimize visualization of fluorescent structures. All image-processing operations were applied uniformly to the entire image stack and were used for visualization purposes only. AVI format renderings were then converted to MP4 format for portability (see Video S1).

## Results

**Figure 1:**
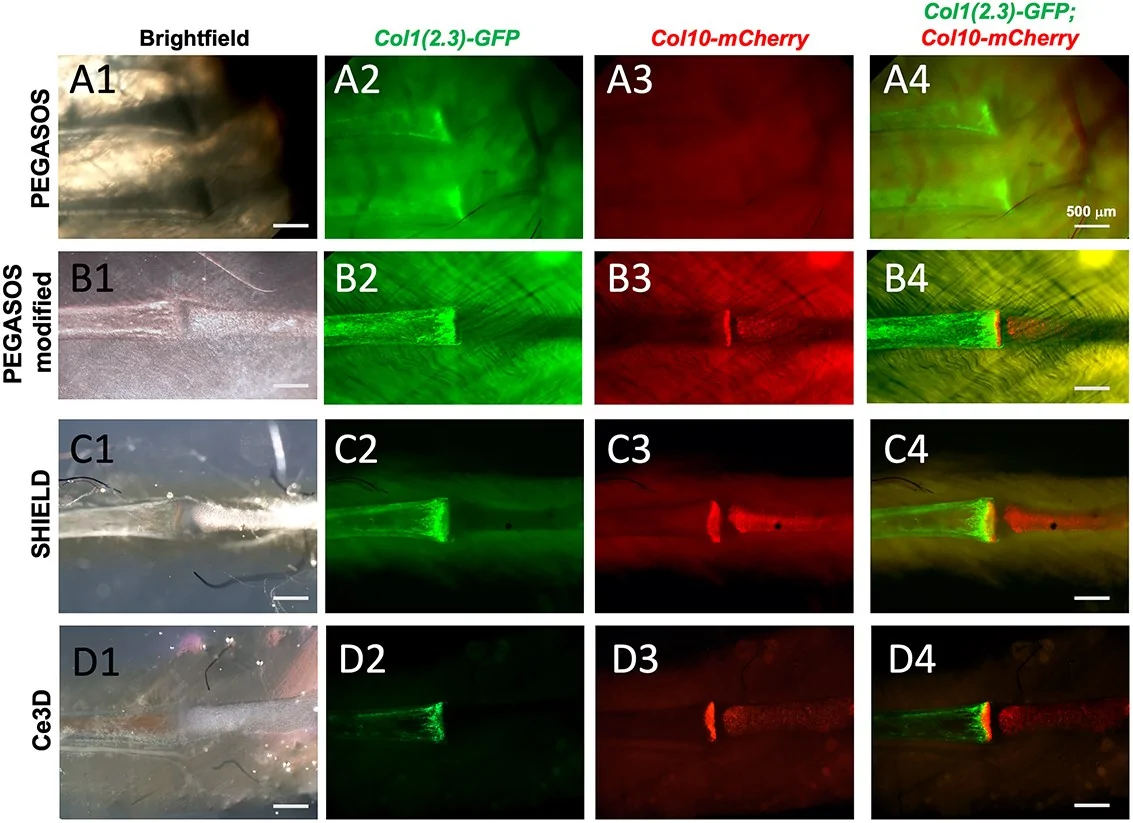
Comparison of tissue clearing methods for preservation of GFP and mCherry fluorescence. Rib samples from Col10-mCherry; Col1(2.3)-GFP double transgenic mice were collected from perfusion-fixed animals and subjected to PEGASOS (A1-A4), a modified PEGASOS protocol with altered temperature and incubation timing (B1-B4), SHIELD (C1-C4), and Ce3D++ (D1-D4) clearing. Samples were imaged using widefield epifluorescence on a Nikon AZ100 macroscope equipped with a Nikon digital sight DS-Fi1 camera. Fluorescent images were adjusted for color levels and minimally sharpened in adobe photoshop. Scale bar = 500 μm.

We tried several methods (Figure 1) before choosing to focus on optimizing the Scale CUBIC protocol. We first tried the PEGASOS method, largely following the published protocol.^5^ In brief, the steps involved perfusion fixation (4% PFA), decolorization (25% Quadrol, at 37 °C), delipidation at 37 °C, dehydration (70% tert-Butanol, 27% PEG, 3% Quadrol, at 37 °C), and index matching (in BB-PEG at 37 °C). While this method resulted in increased tissue transparency, we noticed some loss of GFP signal and substantial loss of mCherry fluorescence (Figure 1, A1-A4). We were able to improve some fluorescence retention by removing the decalcification step, performing the decolorization, delipidation, dehydration, and clearing steps at room temperature instead of 37 °C, and carrying out the dehydration step for 3 d instead of 1. However, while the mCherry signal was better retained, this resulted in a sample that was not as clear (Figure 1, B1-B4). We also tried another method called SHIELD.^16^ We found that this method retained GFP and mCherry signals; however, a high degree of background-fluorescence was evident in muscle tissues (Figure 1, C1-C4). We next tried Ce3D++, a modified version of Ce3D clearing,^15^^,^^21^ which involves two exchanges of one solution and is optimized to match the RI of most microscope oils. This resulted in high clarity; however, we noticed a significant loss in signal for both GFP and mCherry proteins (Figure 1, D1-D4).

**Figure 2:**
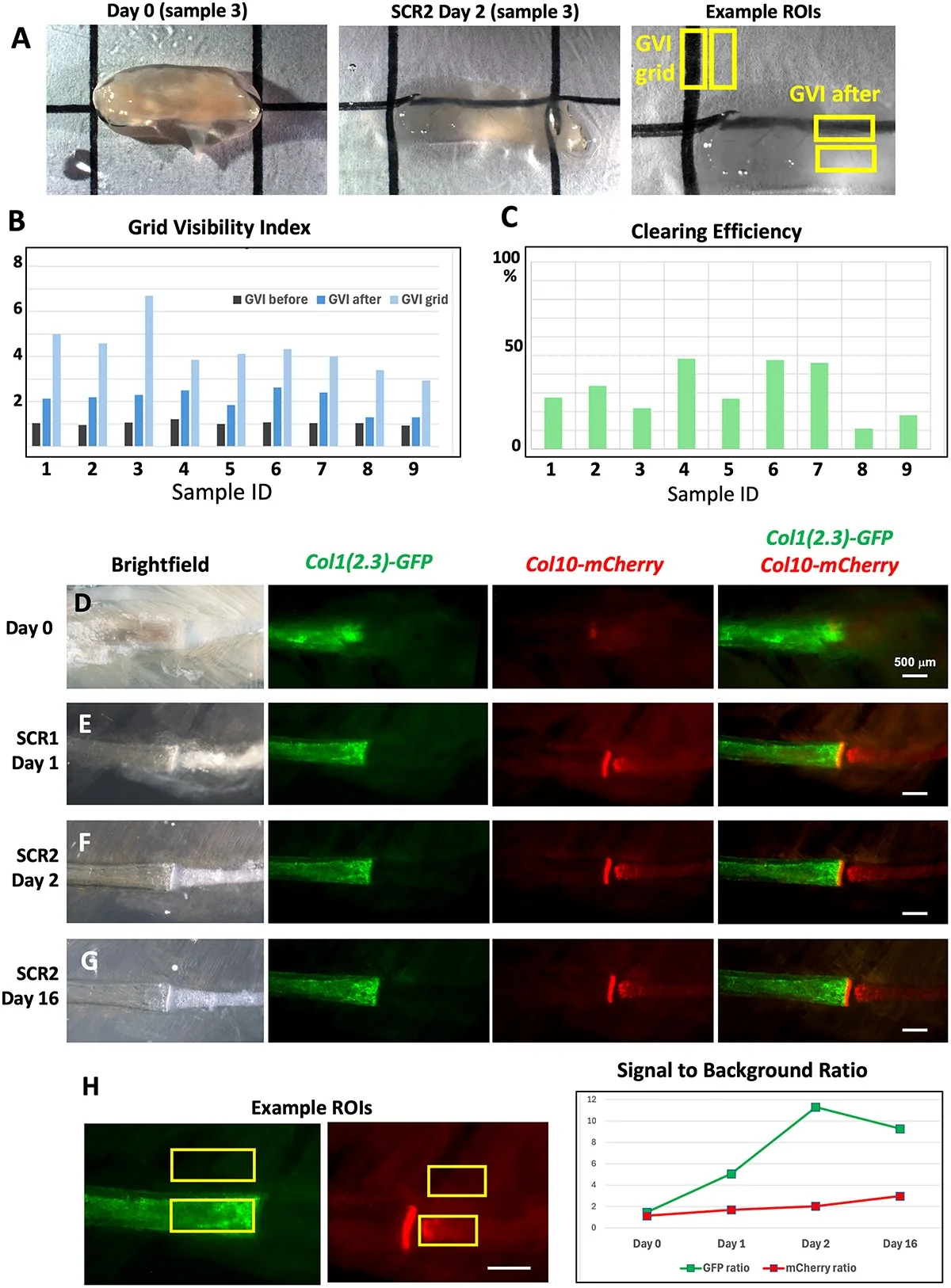
The Scale CUBIC protocol improves optical transparency while preserving GFP and mCherry fluorescence. Rib samples from Col10-mCherry; Col1(2.3)-GFP double transgenic mice were collected from perfusion-fixed animals. Improved transparency was assessed using a grid-visibility assay (A). A representative sample (sample ID = 3) is shown before and after clearing, alongside the corresponding enlarged L* (lightness) channel extracted from the CIELAB image. Yellow rectangles indicate representative ROI used for brightness measurements. These measurements were used to calculate a grid visibility index for uncleared samples, cleared samples, and the unobstructed grid reference (B). The resulting values were subsequently used to determine clearing efficiency (C), where 0% corresponds to the uncleared sample and 100% corresponds to the maximum observable grid contrast measured in the absence of tissue. Samples were also imaged to assess fluorescence at d 0 (D), after immersion in scale CUBIC reagent 1 (SCR1) for 24 h (E, d 1 of the protocol), and after immersion in SCR2 for 24 h (F, d 2 of the protocol). Retention of signal for longer periods (G, d 16) was also assessed. (H) Signal-to-background ratios (SBRs) for GFP and mCherry fluorescence were measured from representative images acquired at d 0, 1, 2, and 16 of the scale CUBIC clearing protocol. SBR was calculated as the mean fluorescence intensity within reporter-positive regions divided by the mean fluorescence intensity of adjacent background regions (see, eg, ROIs). Both GFP and mCherry fluorescence remained readily detectable throughout the clearing process, with SBRs improved following clearing. Quantification was performed on a representative specimen to provide an illustrative numerical assessment of the corresponding image throughout the protocol. Fluorescent images were adjusted for color levels and minimally sharpened in Adobe Photoshop. Scale bar = 500 μm.

We found that instead, an improved Scale CUBIC protocol, rendered rib samples sufficiently optically transparent by effectively reducing light scattering from mineralized and collagen-rich tissue while preserving robust fluorescence of both mCherry and GFP (Figure 2). To quantify tissue transparency using this method, we developed a grid-visibility index based on the visibility of an underlying high-contrast grid before and after clearing (See “Materials and Methods”). Representative images demonstrating progressive visualization of the grid following clearing, consistent with increased optical transparency, are shown in Figure 2A.

Grid visibility index measurements increased following clearing in all samples. Uncleared tissues exhibited GVIs near unity (mean ± SD: 1.04 ± 0.08), indicating minimal visibility of the underlying grid. Following clearing, the mean GVI increased to 2.06 ± 0.51, corresponding to an approximately two-fold increase in grid visibility (Figure 2B). Individual samples exhibited post-clearing GVIs ranging from 1.29 to 2.62. To account for variation in maximum detectable grid contrast, measurements were normalized to images of the unobstructed grid. The unobstructed grid exhibited GVIs ranging from 2.94 to 6.70, reflecting the maximum observable grid contrast under the imaging conditions used. Normalized clearing efficiencies ranged from 10.9% to 48.3%, with a mean clearing efficiency of 31.2 ± 14.1% (Figure 2C). This variability in clearing efficiency is likely related to the thickness and variation in the composition of fat, muscle, and connective tissues in the sample.

Together, these results demonstrate that the modified Scale CUBIC clearing protocol substantially increased optical transparency and improved visualization of underlying structures, as evidenced by increased grid visibility in all samples. By quantifying the visibility of an underlying grid, this method provides a simple, objective, and reproducible measure of tissue transparency that enables direct comparison of clearing efficiency across samples and experimental conditions.

Fluorescence signal was retained following storage in SCR2, with mCherry and GFP fluorescence observed after at least 16 d (shown in Figure 2D-G), demonstrating that the cleared samples remain suitable for delayed imaging and longitudinal analysis. To assess fluorescence preservation during clearing, GFP and mCherry SBRs were measured from representative images acquired throughout the Scale CUBIC protocol. GFP SBRs increased from 1.49 at d 0 to 5.08, 11.32, and 9.30 at d 1, 2, and 16, respectively. Similarly, mCherry SBRs increased from 1.15 at d 0 to 1.71, 2.04, and 2.99 over the same period (Figure 2H). These findings indicate that both GFP and mCherry fluorescence remained detectable throughout the clearing process and that tissue clearing also improved visualization of fluorescent signals relative to background.

**Figure 3:**
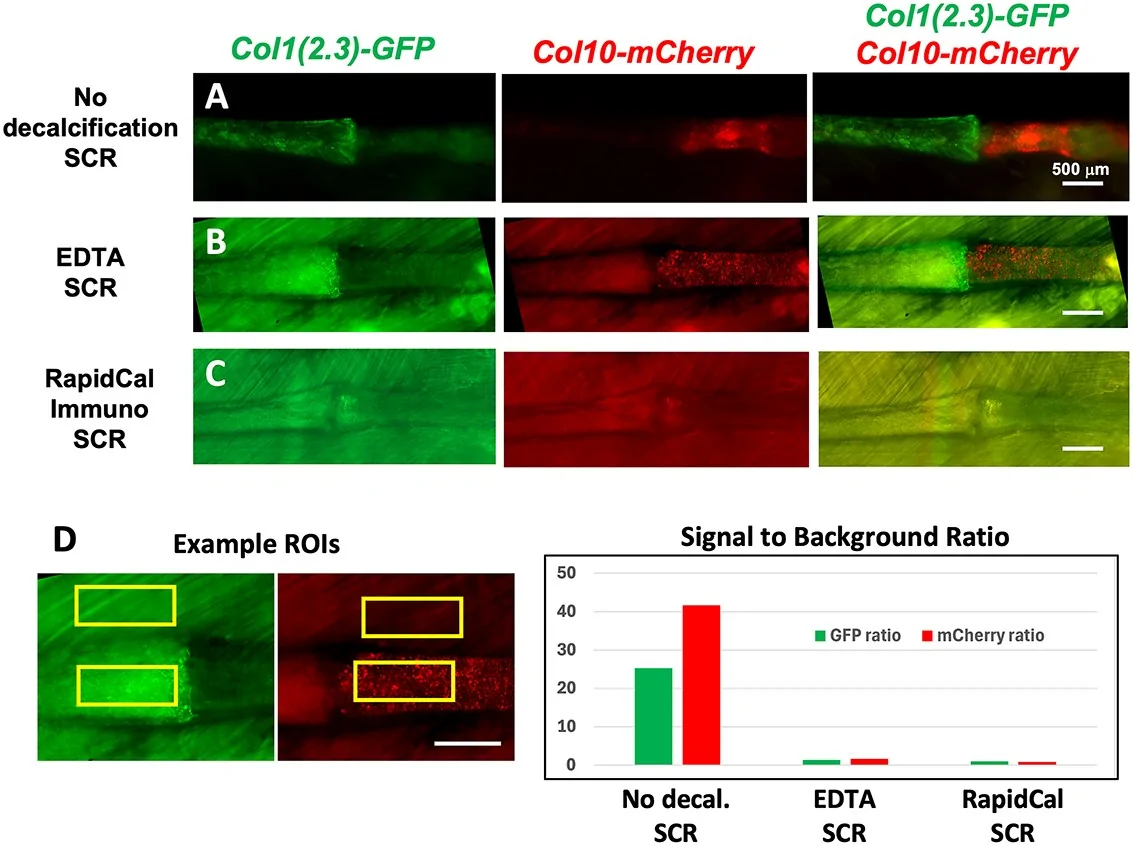
Decalcification resulted in poor resolution of fluorescent signal. (A-C) Samples decalcified using either EDTA (20%) or RapidCal Immuno were cleared with Scale CUBIC and imaged in comparison to samples with no decalcification. (D) GFP and mCherry signal-to-background ratios (SBRs) were measured on a representative specimen to provide an illustrative numerical assessment of the corresponding image. SBR was calculated as the mean fluorescence intensity within reporter-positive regions divided by the mean fluorescence intensity of adjacent background regions (see, eg, ROIs). Both decalcification methods substantially reduced SBRs relative to the non-decalcified Scale CUBIC protocol, consistent with increased diffuse background fluorescence and reduced tissue-specific reporter localization. Measurements were obtained from representative samples and are presented to illustrate the relative effects of the different treatments. Imaging was performed using widefield epifluorescence on a Nikon AZ100 macroscope with a Leica K3C camera. Fluorescent images were adjusted for color levels and minimally sharpened in adobe photoshop. Scale bar = 500 μm.

Using this improved clearing protocol, we next investigated whether decalcification could further enhance tissue transparency (Figure 3). A commonly used decalcification method employs EDTA, which chelates calcium ions from mineralized tissues. Consistent with standard histological processing protocols, samples were decalcified in 20% EDTA at room temperature for 14 d prior to Scale CUBIC clearing. However, following this treatment, all tissues exhibited diffuse fluorescence in both the green and red channels, accompanied by a moderate reduction in mCherry signal within cartilage (Figure 3B).

We then evaluated a more rapid decalcification approach using RapidCal (StatLab RapidCal Immuno Decalcifier, Cat. No. STL6086) for 24-28 h at room temperature prior to Scale CUBIC clearing. This treatment produced an even greater increase in diffuse fluorescence in both channels, resulting in widespread background signal that obscured the tissue-specific localization of the transgenic reporters (Figure 3C).

Using a quantitative assay (See “Materials and Methods”), we compared no decalcification to both EDTA and RapidCal decalcification and observed substantially reduced signal specificity (Figure 3D). GFP SBRs decreased from 25.3 in Scale CUBIC-cleared tissues to 1.36 and 1.07 following EDTA and RapidCal treatment, respectively. Similarly, mCherry SBRs decreased from 41.7 to 1.65 and 0.85 following EDTA and RapidCal treatment, respectively. These findings quantitatively support the observed increase in diffuse background fluorescence and loss of tissue-specific reporter localization following decalcification. Consequently, neither decalcification protocol improved imaging outcomes, both adversely affected the specificity of fluorescent signal detection.

**Figure 4:**
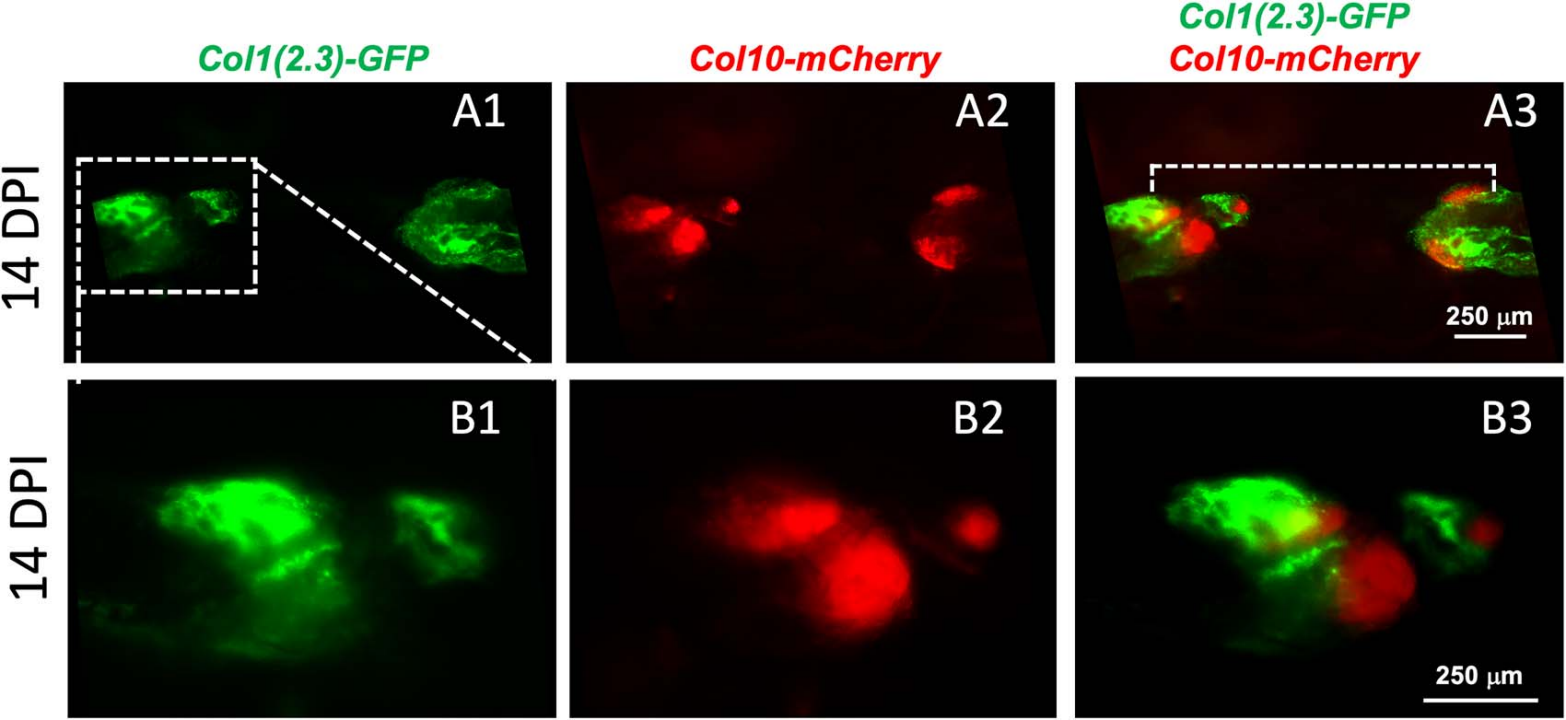
Application of Scale CUBIC clearing to rib bone following skeletal injury. A rib sample from a *Col10-mCherry; Col1(2.3)-GFP* double transgenic mouse that underwent a bone resection (~3 mm), was collected from a perfusion-fixed animal 14 d post injury and cleared using the our Scale CUBIC clearing protocol. Images of the resection site were acquired following immersion in Scale CUBIC reagent 2 using a Leica THUNDER imaging system with the Large Volume Computational Clearing (LVCC) algorithm and a 5× objective (A1-A3, dashed lines in A3 indicate the extent of the resection). Higher magnification views are shown from one of the cut ends (dashed lines indicate the region) to visualize dual reporter fluorescence. Images were adjusted for color levels and minimally sharpened in Adobe Photoshop. Scale bars = 250 μm.

We next evaluated our method in a rib resection injury model, which we have previously shown induces the formation of a repair callus enriched in cells expressing osteogenic and chondrogenic markers, particularly at the resection margins (Figure 4).^22^ By preserving fluorescence signal, Scale CUBIC enabled detailed visualization of the cellular composition and spatial architecture of the reparative tissue.

**Figure 5:**
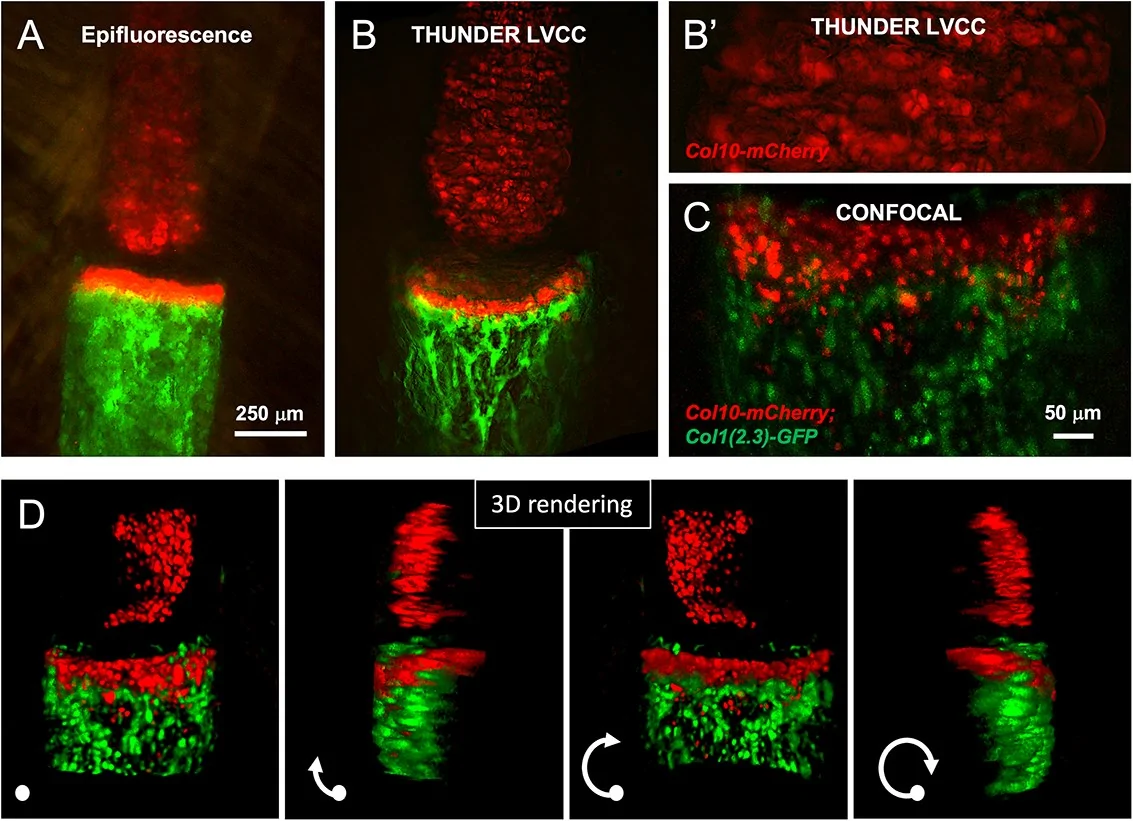
High-resolution imaging of cleared rib growth plate and costal cartilage using computational and optical sectioning. To resolve individual cells, cleared rib samples were imaged at higher magnification using the Nikon AZ100 widefield microscope (A), the Leica THUNDER imaging system with LVCC (B, B′), and confocal microscopy (Leica SP8X; maximum intensity projection of a *z*-stack spanning ~300 μm in depth) (C). These complementary imaging approaches enabled visualization of cellular architecture within the growth plate and costal cartilage of intact tissues. Fluorescence images were uniformly adjusted for brightness and color levels in Adobe Photoshop for display purposes. Scale bars = 250 μm or 50 μm, as indicated. (D) A 3D rendering was generated (see Video S1) and representative frames were extracted and are shown to illustrate the spatial organization of fluorescently labeled structures throughout the tissue volume.

Low-magnification imaging of cleared ribs can be performed using standard compound or stereomicroscopes equipped with widefield fluorescence optics; however, higher-magnification analysis requires optical or computational sectioning to resolve cellular detail within thick samples. Accordingly, high-resolution imaging was best achieved using specialized microscopy platforms, including confocal microscopy or computational clearing systems (Figure 5). For computational clearing, we used the LVCC algorithm on the Leica THUNDER imaging system to suppress out-of-focus light and enhance contrast throughout the tissue volume. Using confocal microscopy, we achieved imaging depths of approximately 300 μm, sufficient to capture cellular organization within the growth plate with both a MIP (Figure 5C) and 3D rendering (Figure 5D, see Video S1) while maintaining a strong SBR.

## Discussion

Optical clearing of mineralized tissues remains challenging due to the combined effects of dense extracellular matrix, mineralized tissue, and autofluorescence, all of which limit light penetration and reduce image quality. Although numerous hard-tissue clearing methods have been developed, many achieve increased transparency at the expense of endogenous fluorescent protein preservation, require decalcification that alters tissue composition, or involve lengthy and technically demanding workflows. In this study, we demonstrate that modest modifications to the Scale CUBIC protocol provide a practical balance between tissue transparency and fluorescence preservation, enabling rapid clearing of adult mouse rib tissue without decalcification while maintaining robust GFP and mCherry fluorescence.

A major advantage of this approach is the elimination of the decalcification step. Decalcification is frequently incorporated into hard-tissue clearing workflows to reduce light scattering from the mineralized matrix and improve transparency. However, our results indicate that this benefit may come at the cost of fluorescence preservation and signal specificity. Both EDTA and RapidCal decalcification produced increased diffuse background fluorescence and markedly reduced GFP and mCherry SBRs, substantially impairing the ability to resolve tissue-specific reporter localization. In contrast, our modified Scale CUBIC protocol achieved sufficient transparency for imaging while preserving endogenous fluorescent signals and their spatial distribution within bone and cartilage. These findings suggest that, at least for mouse rib tissues, useful imaging depth can be achieved through optimized delipidation and RI matching alone, without chemical removal of mineral content. Nevertheless, skeletal elements with substantially greater mineral density may require additional clearing strategies, representing an important limitation of the current approach.

Preservation of endogenous fluorescence was a primary objective of protocol development. Through evaluation of several clearing approaches, we found that a modified Scale CUBIC protocol provided the most favorable combination of transparency, low background fluorescence, and retention of both GFP and mCherry signals. This was particularly important for mCherry, which is known to be sensitive to changes in pH, temperature, and chemical environment. We found that reducing the triethanolamine concentration in SCR2 and performing all clearing steps at room temperature were critical for maintaining red fluorescent protein fluorescence, emphasizing that relatively small modifications to reagent composition and incubation conditions can have substantial effects on fluorophore preservation. Quantitative analysis further demonstrated that GFP and mCherry fluorescence remained readily detectable throughout the clearing process and during extended storage in SCR2, supporting the use of this protocol for delayed imaging, reimaging, and batch processing of samples.

Application of our protocol to a rib resection model further demonstrated its utility in a biologically relevant setting. Preservation of Col10-mCherry and Col1(2.3)-GFP fluorescence enabled visualization of cellular organization within the repair callus, particularly near resection margins where osteogenic and chondrogenic lineage cells are enriched. These observations highlight the value of fluorescence-preserving clearing methods for studying skeletal development, repair, and regeneration, where maintaining spatial relationships between distinct cell populations is often essential.

Despite these advantages, several limitations should be noted. First, this protocol was improved and validated using GFP and mCherry reporters; its compatibility with other fluorescent proteins or chemical fluorophores remains to be determined and may require further modification of reagent composition or imaging conditions. Second, while the protocol performed well in adult mouse ribs, clearing efficiency and imaging depth will likely be reduced in larger and more heavily mineralized skeletal elements. Finally, although the protocol enabled confocal imaging to depths of approximately 300 μm, image quality progressively declined with depth due to the combined effects of light attenuation, optical aberrations, and residual tissue scattering. Future studies may explore modified reagent formulations, longer clearing durations, alternative RI matching solutions, or partial decalcification approaches to extend transparency and thus imaging depth in larger skeletal tissues.

In summary, we present a modified Scale CUBIC protocol that enables rapid clearing of mouse rib bone and cartilage while preserving endogenous fluorescent protein signals and avoiding decalcification. By balancing tissue transparency with fluorescence retention and structural preservation, this approach provides a practical method for imaging mineralized tissues in fluorescent reporter mouse models and should be readily adaptable for studies of skeletal biology, repair, and regeneration.

## Supplementary Material

Col1(2_3)-GFP_Col10-mCherry_ziag122

## Data Availability

Measurements for graphs in Figures 2 and 3 are available on request and can also be found at https://bit.ly/4e0FzOw.
